# Inhibition of *Porphyromonas gingivalis* peptidyl arginine deiminase, a virulence factor, by antioxidant-rich *Cratoxylum cochinchinense*: *In vitro* and *in silico* evaluation

**DOI:** 10.1016/j.sjbs.2021.12.037

**Published:** 2021-12-17

**Authors:** Sheri-Ann Tan, Hok Chai Yam, Siew Lee Cheong, Yoke Chan Chow, Chui Yin Bok, Jia Min Ho, Pei Yin Lee, Baskaran Gunasekaran

**Affiliations:** a Department of Bioscience, Faculty of Applied Sciences, Tunku Abdul Rahman University College, Jalan Genting Kelang, 53300 Setapak, Kuala Lumpur, Malaysia; b Department of Biotechnology, Faculty of Applied Sciences, UCSI University, 56000 Cheras, Kuala Lumpur, Malaysia; c Department of Pharmaceutical Chemistry, School of Pharmacy, International Medical University, 57000 Bukit Jalil, Kuala Lumpur, Malaysia

**Keywords:** *Cratoxylum cochinchinense*, *Porphyromonas gingivalis*, Peptidyl arginine deiminase, Anti-citrullination, Mangiferin, Vismiaquinone A, ABTS, 2,2′-Azino-bis (3-ethylbenzthiazoline-6-sulfonic acid) diammonium salt, ACPA, Anti-citrullinated peptide antibodies, BAEE, Benzoyl-L-arginine ethyl ester, DNA, Deoxyribonucleic acid, DPPH, 2,2-diphenyl-1-picrylhydrazyl, DTT, Dithiothreitol, EDTA, Ethylenediamine tetraacetic acid, FRAP, Ferric reducing antioxidant power, GAE, Gallic acid equivalence, HPLC, High performance liquid chromatography, IPTG, Isopropyl β- d-1-thiogalactopyranoside, OPLS, Optimized potentials for liquid simulations, PAD, Peptidylarginine deiminase, PCR, Polymerase chain reaction, PDB, Protein data bank, PMSF, Phenylmethylsulfonyl fluoride, PPAD, Porphyromonas gingivalis peptidylarginine deiminase, QE, Quercetin equivalence, RA, Rheumatoid arthritis, SD, Standard deviation, SDS-PAGE, Sodium dodecyl sulphate–polyacrylamide gel electrophoresis, TFC, Total flavonoid content, TPC, Total phenolic content, TPTZ, 2,4,6-Tripyridyl-S-triazine

## Abstract

*Porphyromonas gingivalis*, the cause of periodontitis, is also linked to many systemic disorders due to its citrullination capability from a unique peptidyl arginine deiminase (PPAD). Protein citrullination is able to trigger an autoimmune response, increasing the severity of rheumatoid arthritis. The main objective of this study is to evaluate the inhibitory activity of *Cratoxylym cochinchinense* leaves extract towards the PPAD *in vitro* and *in silico*. Methanolic extract of *Cratoxylum cochinchinense* (CCM) was tested for total phenolic and flavonoid contents along with antioxidative assays. Inhibition of PPAD activities was conducted thereafter using recombinant PPAD in cell lysate. Phytocompounds postulated present in the CCM such as mangiferin, vismiaquinone A, δ-tocotrienol and α-tocotrienol and canophyllol were used as ligands in a simulated docking study against PPAD. Results obtained indicated high antioxidant potential in CCM while recording abundant phenolic (129.0 ± 2.5495 mg GA/g crude extract) and flavonoid (159.0 ± 2.1529 mg QE/g crude extract) contents. A dose-dependent inhibition of PPAD was observed when CCM was evaluated at various concentrations. CCM at 1 mg/mL exhibited citrulline concentration of 24.37 ± 3.25 mM which was 5 times lower than the negative control (114.23 ± 3.31 mM). Molecular docking simulation revealed that mangiferin and vismiaquinone A engaged in H-bonding and pi-pi interactions with important active site residues (Asp130, Arg152, Arg154 and Trp127) of PPAD and could be the potential phytochemicals that accounted for the inhibitory activities observed in the methanolic leaves extract. As such, CCM could be further explored for its therapeutic properties not only for periodontitis, but also for other systemic diseases like rheumatoid arthritis.

## Graphical abstract

**Figure:**
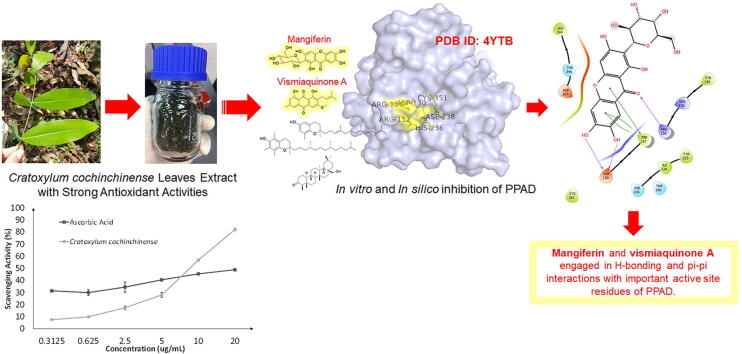

## 1 Introduction

*Porphyromonas gingivalis* is an obligate anaerobe residing within the oral cavity and is the main causal agent of periodontitis. The persistence of this microbe in the host cell is perpetuated by the presence of virulence factors such as lipopolysaccharides, fimbriae (FimA and Mfa1), hemagglutinins, gingipain proteases and a peptidyl arginine deiminase (PPAD) (Lamont et al., 2018). PPAD enables *P. gingivalis* to citrullinate C-terminal as well as free arginines, a simple process that increases its chances of survival within the host cell environment (Vermilyea et al., 2019). The amino acid conversion process allows *P. gingivalis* to evade human innate immunity (Stobernack et al., 2018) besides playing a role in biofilm formation (Vermilyea et al., 2019, Karkowska-Kuleta et al., 2018) and cell translocation (Vermilyea et al., 2021). Furthermore, citrullination is found to be the link between this pathobiont and other systemic disorders especially rheumatoid arthritis (RA) (Lee et al., 2019, Gómez-Bañuelos et al., 2019). In rheumatoid arthritis, PPAD works in concert with another virulence protein, the gingipain proteases. Gingipains cleave proteins and expose arginine residues for PPAD-mediated citrullination leading to production of neoantigens (Singhrao and Olsen, 2019). While citrullination is a regulated post-translational modification carried out by human peptidyl arginine deiminase 4 (PAD4) in our body, gingipain assisted-PPAD deregulates citrullination of peptides to instigate the production of anti-citrullinated protein antibody (ACPA). PPAD has been known to citrullinate vimentin, α-enolase, and fibrinogen (Sakaguchi et al., 2019, Maldonado et al., 2020), which are all known autoantigens of RA that are targeted by ACPA (Jenning et al., 2020). In the past, inhibition of gingipains by synthetic compounds had demonstrated positive outcome. However, these molecules proved unsuitable in *in vivo* studies due to their interference with important host proteolytic enzymes (Olsen and Potempa, 2014). Hence, inhibitors from natural sources especially from plants are much promising in this regard. Phytocompounds shown to inhibit gingipains include *Prunus cerasus* L. phenolic extract (Ben Lagha et al., 2021), argeloside I isolated from *Solenostemma argel* leaves (Eltigani et al., 2020) and Ginger exosome-like nanoparticles (GELN) extracted from ginger plants (Sundaram et al., 2019).

Nevertheless, a recent paper suggested PPAD as a more credible inhibitory target since it was found to regulate the biogenesis of gingipain-containing outer membrane vesicles as well as modulating the proteolytic activities of gingipains (Vermilyea et al., 2021). Hence, inhibition of PPAD indirectly affects gingipains as well, thus, killing two birds with one stone. To date, therapeutic inhibitors for PPAD have yet to be identified and efforts to synthesize and develop inhibitors for the enzyme have also not been reported.

*Cratoxylum cochinchinense* is a native species in Southeast Asia, under the family Hypericaceae. This plant is a traditional medicinal plant of which its roots, barks, twigs and leaves can also be utilized to treat minor illnesses such as fevers, burns and abdominal complications (Thu et al., 2017). In terms of phytochemicals, *C. cochinchinense* contains abundance of phenolic compounds such as xanthones (Li et al., 2018, Tang et al., 2004), anthraquinones, tocotrienols (Chailap et al., 2017) and triterpenoids (Lv et al., 2019). Plant secondary metabolites especially phenolic compounds have demonstrated various biological actions to sustain human health through direct interaction with cellular proteins (Fraga et al., 2010). Studies have also shown the inhibitory effects of these molecules towards both eukaryotic (Martinez-Gonzalez et al., 2017) and prokaryotic enzymes (Ahmad et al., 2012). Furthermore, xanthones, are reported to exert high antioxidant activities (Gunter et al., 2020) besides being able to inhibit *Porphyromonas gingivalis* biofilm formation (Widyarman et al., 2019). Importantly, *Cratoxylum formosum*, a close relative of *Cratoxylum cochinchinense*, is widely used in periodontal research and both these species share similar phytochemical profiles (Son, 2020). Therefore, in this work, we investigated the antioxidant as well as PPAD inhibitory activities of the leaves extract of *Cratoxylum cochinchinense* through *in vitro* and *in silico* methods.

## 2 Materials and methods

### 2.1 Chemicals and reagents

All chemicals and solvents used were of analytical or HPLC grade. Ascorbic acid, Folin-Ciocalteu’s phenol reagent, gallic acid, calcium chloride, perchloric acid 70%, ammonium iron (III) sulfate dodecahydrate, ferrous ammonium sulfate hexahydrate and *ortho*-phosphoric acid 99% were purchased from Merck (Germany). Other chemicals such as 2,2′-Azino-bis (3-ethylbenzthiazoline-6-sulfonic acid) diammonium salt (ABTS) reagent, 2,2-diphenyl-1-picrylhydrazyl (DPPH) reagent, Ferrozine, Trolox, quercetin, Nα-benzoyl-L-arginine ethyl ester (BAEE), DL-Dithiothreitol (DTT), 2,3-butanedione monoxime, L-citrulline, Bradford reagent and chloro-amidine were procured from Sigma-Aldrich (USA).

### 2.2 Sample collection, preparation and extraction

*Cratoxylum cochinchinense* was obtained from a local farm, Herbal Oasis, located in Negeri Sembilan, Malaysia. The plant was identified and the voucher specimen (Voucher No.: MFI 0229/21) was deposited at Herbarium, Biodiversity Unit, Universiti Putra Malaysia. The leaves of *C. cochinchinense* were cleansed with distilled water. The samples were air-dried for 24 h and dried in oven at 40 °C for 2 weeks. The dried leaves were ground into fine powder and stored under 4 °C. The extraction was done by macerating 200 g of dried leaves in 1:10 (w/v) ratio of 80% methanol. After 48 h, approximately 200 mL of the 80% methanol was collected and 500 mL of fresh 80% methanol was then added. This process was repeated every two days and the extract solvents were collected on the ninth day of maceration. The extract solvent was filtered and concentrated by rotary evaporator. The concentrated extract was dried in oven at 40 °C for 3 days to remove excess methanol and frozen dried. After that, methanolic extract of *Cratoxylum cochinchinense* (CCM) was stored in −20 °C freezer. The extract was dissolved in absolute methanol and diluted into desired concentrations for the subsequent experiments.

### 2.3 *In vitro* antioxidant activities of CCM

#### 2.3.1 Total phenolic (TPC) and flavonoid contents (TFC)

TPC and TFC were obtained based on Folin-Ciocalteau and aluminium chloride colorimetric procedures, respectively (Stanković, 2011) using 2.5 mg/mL of CCM. Results were expressed in Gallic Acid Equivalence (mg GAE/ g of dry extract) for total phenolic contents and Quercetin Equivalence (mg QE/ g of dry extract) for total flavonoid contents.

#### 2.3.2 DPPH and ABTS radical scavenging assays

For DPPH radical scavenging assay, a total of 3 mL sample was added into 1 mL of 0.1 mM DPPH reagent according to Shen et al. (2010). The mixture was incubated in the dark for 30 min before measuring the absorbance at 517 nm. The procedure for ABTS assay was based on Lee et al. (2015) with minor modifications. The photometric assay was conducted by mixing 100 µl of the ABTS working reagent to 100 µl of the plant extract in a 96-well microtiter plate and then incubated for 6 min at room temperature. After incubation, absorbance was measured for each reaction mixture at 734 nm. For blank control, the sample was substituted with absolute methanol; for positive control, Trolox was used. The radical scavenging activity was calculated by using the formula below:

$$\mathrm{Scavenging}\;activity\;\left(\%\right)=\left[\left(A_{\mathrm{control}}-A_{s\mathrm{ample}}\right)/A_{\mathrm{control}}\right]\times 100\%$$

wherein,

A_*control*_: Absorbance reading of control group and A_*sample*_: Absorbance of sample group

#### 2.3.3 Ferric reducing antioxidant power (FRAP) and ferrous ion chelating assays

Ferric reducing antioxidant power was determined using the method by Benzie and Strain (1999). FRAP working reagent was mixed with 0.1 mL of plant extract. The mixture was resuspended vigorously and left for 30 min incubation at room temperature. The absorbance of the reaction mixture was then determined at 593 nm using a UV–Vis spectrophotometer (Hitachi U-2900, Japan). A calibration curve was plotted using ferrous sulphate. The results were expressed as ferrous sulphate equivalence (mg FeSO_4_/ g of dry extract). Ferrous ion chelating activity was conducted based on a method described by Chew et al. (2009). Briefly, 1 mL of plant extract was added with 1 mL of 0.1 mM ferrous sulphate (FeSO_4_) followed by 1 mL of 0.25 mM Ferrozine. The reaction mixture was incubated for 10 min in the dark at room temperature. Then, the absorbance of each reaction mixture was measured at 562 nm using UV–Vis spectrophotometer (Hitachi U-2900, Japan). The ferrous ion chelating activity was calculated by using the formula below:

$$\mathrm{Ferrous}\;\mathrm{ion}\;\mathrm{chelating}\;\mathrm{activity}\;\left(\%\right)=\left[\left(A_{\mathrm{control}}-\;A_{\mathrm{sample}}\right)/A_{\mathrm{control}}\right]\times 100\%$$

Wherein,

A_*control*_: Absorbance reading of control group and A_*sample*_: Absorbance of sample group

### 2.4 Inhibition of PPAD citrullination by CCM

#### 2.4.1 Recombinant PPAD preparation

A truncated *Porphyromonas gingivalis* peptidyl arginine deiminase (PPAD) gene encoding amino acid 49–484 in pUCIDT plasmid was commercially obtained from Integrated DNA Technologies through gene synthesis. The truncated PPAD was found to have higher solubility and citrullination activity (Montgomery et al., 2016). The PPAD gene was PCR-amplified using a pair of primers tagged with complementary sequences to the pET47 plasmid to allow homologous recombination cloning (Jacobus and Gross, 2015). The PCR-amplified pET47 plasmid was subjected to DpnI digestion and purified. Both PPAD and pET47 DNA fragments harboured the same 20 nucleotide sequences at their 5′ and 3′ termini. A reaction mix comprising 1:5 ratio of pET47 plasmid to PPAD insert and 5 × KCM buffer (Chung and Miller, 1993) were transformed into DH5α *Escherichia coli* competent cells and selected on Luria-Bertani (LB) agar plates containing 100 µg/ml ampicillin (Hanahan, 1983). Colony PCR screening was performed to identify positive transformants using T7 promoter and terminator sequencing primers. The positive clone candidates were further verified by DNA sequencing. Subsequently, the plasmid harbouring PPAD gene was retransformed into BL21 (DE3) *E. coli* cells for protein expression. PPAD expression was induced with 1 mM isopropyl β-d-1-thiogalactopyranoside (IPTG) when the cell density achieved 0.6 measured at 600 nm. Upon induction at 37 °C for three hours, the bacterial cells were harvested and resuspended in 10 mL buffer (25 mM HEPES, 150 mM NaCl, 0.5 mM PMSF) followed by sonication. The expression of PPAD were then assessed by 10% SDS-PAGE.

#### 2.4.2 Reaction mixture preparation

Reaction buffer was prepared by the addition of 10 mM DL-dithiothreitol (DTT), 10 mM CaCl_2_ and 100 mM Tris buffer at final volume of 400 µl. 200 µg of recombinant BL21 (DE3) PPAD lysate was added to the buffer. Then, plant extract (1 mg/mL, 0.75 mg/mL, 0.5 mg/mL, 0.25 mg/mL) was pipetted into the reaction while Cl-amidine (50 µM final concentration) was used as positive control. The mixture was incubated at 37 °C for 2 min to prewarm the solution. The reaction was activated with 100 µl 100 mM Nα-benzoyl-L-arginine ethyl ester (BAEE) and the tubes were incubated at 37 °C for 30 min. Perchloric acid was used to terminate the reaction. Samples were centrifuged at 12,000 rpm for 15 min at 24 °C (Teo et al., 2012).

#### 2.4.3 Citrulline colorimetric test

Briefly, 200 µl Redox reagent was added to the samples for colour development. The solution was boiled for 10 min and left to cool at room temperature. Butanedione monoxime was then added to the samples. Absorbance of the reaction mix was measured at 490 nm wavelength using a spectrophotometer. The concentration of citrulline produced was obtained based on the citrulline standard curve (Teo et al., 2012).

### 2.5 Molecular docking of selected ligands against PPAD

**Fig. 1:**
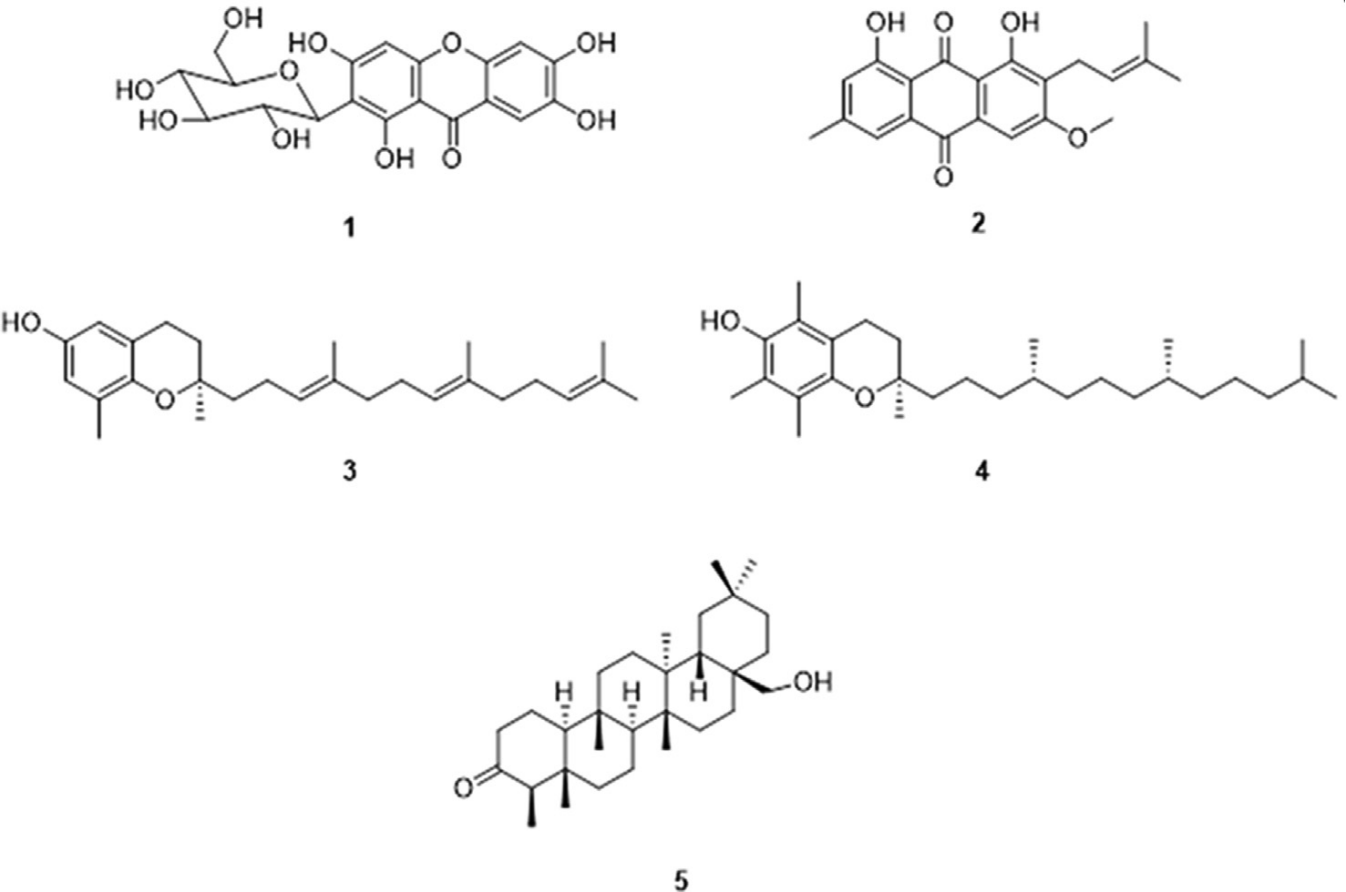
Phenolic compounds [mangiferin (**1**), vismiaquinone A (**2**), δ-tocotrienol **(3**), α-tocotrienol (**4**)] and triterpene [canophyllol (**5**)] previously found in the leaves extract of *Cratoxylum cochinchinense*.

Molecular docking simulation of 5 main phytocompounds (Fig. 1), namely mangiferin (**1**), vismiaquinone A (**2**), δ-tocotrienol (**3**), α-tocotrienol (**4**) and canophyllol (**5**) (Lv et al., 2019, Chailap et al., 2017, Tang et al., 2004) postulated present in the leaves extract of *Cratoxylum cochinchinense* was performed on the *Porphyromonas gingivalis* peptidylarginine deiminase (PPAD) (PDB ID: 4YTB) (Goulas et al., 2015) via Schrödinger modeling software (Maestro version 12.7) (Maestro, 2021). Structure-cleaning step utilizing (LigPrep, 2021) was carried out to convert two-dimensional structures of the compounds to three-dimensional, to generate stereoisomers, and to determine the most probable ionization state at pH 7. Conformers for each compound was generated through ConfGen by applying the OPLS-2005 force field method. The crystal structure of the PPAD was downloaded from the Protein Data Bank and prepared using the Protein Preparation Module (Protein Preparation Wizard, 2021) in Maestro. Crystallographic waters were removed. Hydrogen bonding networks was automatically optimized, and the resultant protein structure was energy-minimized prior to docking. Docking grid was centred on the binding site of reference compound, aspartate-glutamine dipeptide with grid coordinates of x: −12.69, y: −7.22, z: −5.92. The grid box encompassed the active site residues consisting of Asp130, Arg152, Arg154, Tyr233, His236, Asp238, Asn297 and Cys351. The docking calculations were carried out using Glide Standard Precision (SP) protocol (Friesner et al., 2004). Binding poses and interactions of each compound with adjacent residues of binding pocket were analyzed.

### 2.6 Statistical analysis

The results obtained for each assay was analysed by IBM SPSS version 21.0. The data were tested for homogeneity of variances by Levene test. For multiple comparisons, one-way analysis of variance (One-way ANOVA) was performed followed by Tukey test when variances are homogenous and Tamhane test when variances are not homogenous. Paired *t*-test was employed to compare two sample groups for DPPH and ABTS assays. All data were tested at the level of significance where p < 0.05.

## 3 Results

### 3.1 *In vitro* antioxidant activities of CCM

**Fig. 2:**
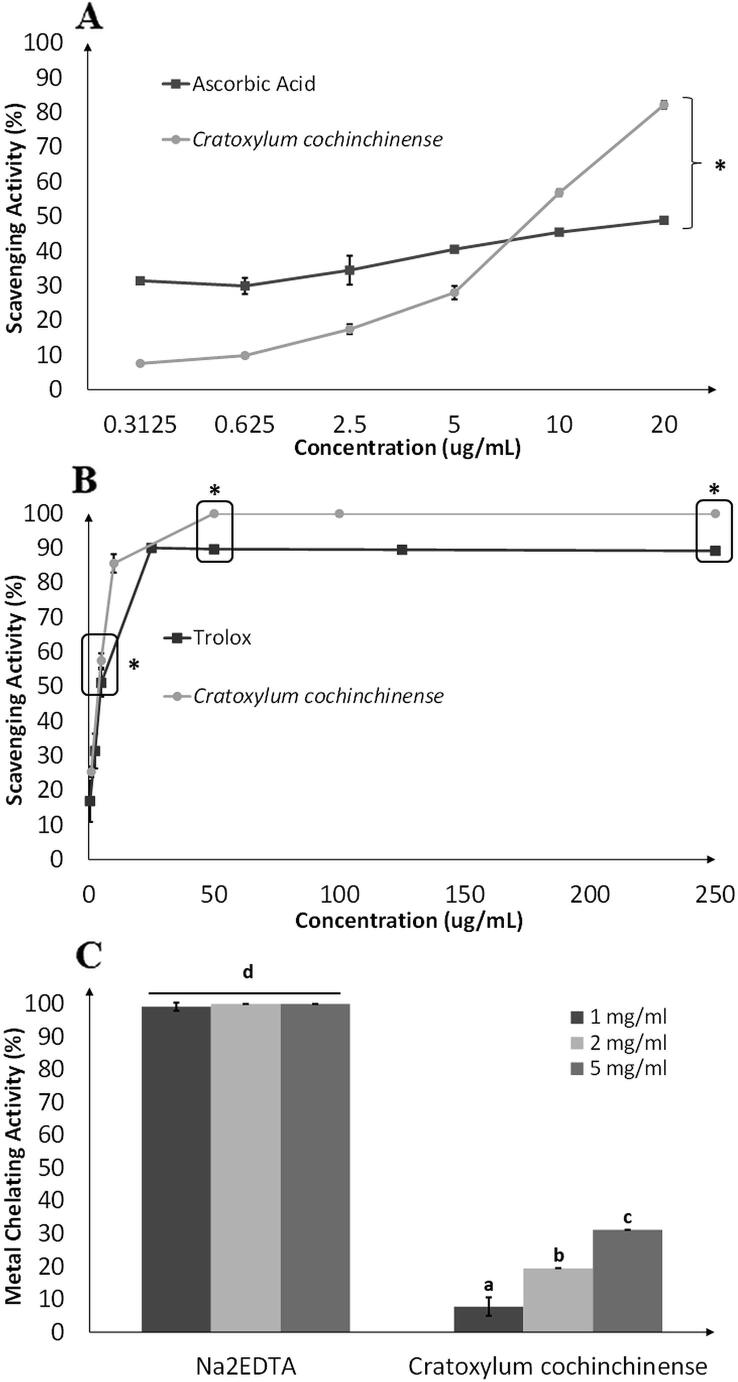
DPPH (A) and ABTS (B) free radical scavenging activities and metal chelating potential (C) of *Cratoxylum cochinchinense* methanolic extract at different concentrations. Ascorbic acid, Trolox and EDTA are positive controls. Values are expressed as means ± SD. Asterisk represents significant difference between samples at similar concentration, p < 0.05 (A and B). Bars with alphabets a, b, c and d are significantly different from each other, p < 0.05. On the contrary, Na_2_EDTA across all concentrations tested are not significantly different as indicated by only alphabet d (C).

The methanolic leaves extract of *Cratoxylum cochinchinense* (CCM) was shown to possess high contents of phenolics and flavonoids with values amounting to 129.0 ± 2.5495 mg GAE/g crude extract and 159.0 ± 2.1529 mg QE/g crude extract, respectively. Furthermore, it was clearly demonstrated that this plant extract exhibited significantly stronger DPPH radical scavenging property as compared to the positive control (ascorbic acid) at 10 μg/mL onwards. A dose-dependent scavenging activities were observed in CCM at tested concentrations ranging from 0.3125 μg/mL to 20 μg/mL (Fig. 2 **(A)**). Similarly, CCM was found to be an effective ABTS radical scavenger as well, outperforming the positive control, Trolox, which was a water-soluble analogue of Vitamin E. The ABTS scavenging potential of CCM reached maximum potency at the concentration of 50 μg/mL (Fig. 2 **(B)**). Besides, CCM also reported a ferric reducing antioxidant power (FRAP) value of 99.33 ± 13.28 mg Fe (II)/g crude extract indicating that this extract should contain reducing agents to convert ferric ions into its ferrous form. Nevertheless, the methanolic extract exerted slightly weaker metal chelating activity of 31% when examined even at a high concentration of 5 mg/mL (Fig. 2 **(C)**).

### 3.2 Inhibition of PPAD citrullination by CCM

**Fig. 3:**
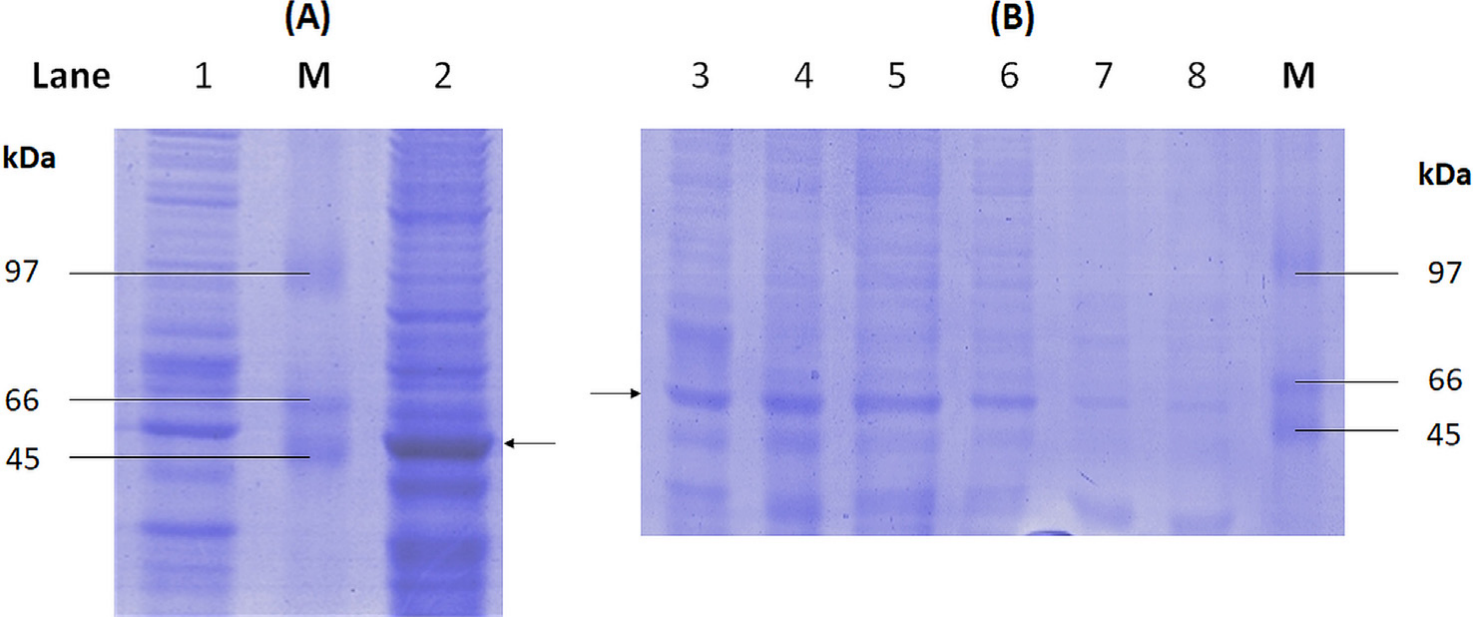
10% SDS-PAGE analysis of the recombinant PPAD protein. Expression profile study. Lane 1 contained the uninduced sample (t = 0, negative control). Lane 2 contained the induced protein harvested at 3 h (A). Solubility of PPAD protein. Lane 3 to 6 contained soluble fractions, insoluble fractions are in Lane 7 and 8 (B). The arrows in the gels indicate the protein band of about 50 k Da corresponding to the molecular weight of recombinant PPAD protein. Lane M, protein markers in kDa.

The PCR product of codon-optimised PPAD gene (1351 bp) was purified and subcloned in frame with ATG initiation codon of the pET-47b (+) expression vector under control of T7 promoter. The nucleotide sequence was verified by DNA sequencing prior protein expression. In Fig. 3**(A)**, a more intense 50 kDa protein band of recombinant PPAD was observed after 3 h of IPTG induction compared to the uninduced sample indicating the successful expression in BL21 (DE3) *Escherichia coli* host cell. After sonication and centrifugation of the bacteria, total protein, supernatants, and inclusion bodies were separated with 10% SDS-PAGE. As shown in Fig. 3**(B)**, the expression strain produced the recombinant protein preferably in soluble form at the predicted molecular weight of 50 kDa.

**Fig. 4:**
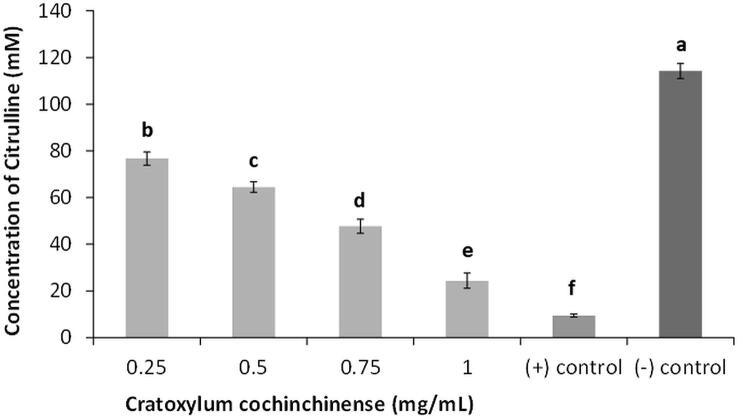
Reduction of citrullination after treatment with *Cratoxylum cochinchinense* methanolic extract. Positive control: Cl-amidine (50 µM). Values are expressed as means ± SD. Bars with alphabets a, b, c, d, e and f are significantly different from each other, p < 0.05.

This cell lysate containing the recombinant PPAD was then used to determine the inhibitory effect of CCM against the citrullination activity of PPAD. Interestingly, CCM revealed inhibition properties towards PPAD in a dose dependent manner when tested at concentrations ranging from 0.25 mg/mL to 1 mg/mL (Fig. 4). The plant extract at 1 mg/mL exhibited citrulline concentration of 24.37 ± 3.25 mM which was 5 times lower than the negative control (114.23 ± 3.31 mM). Nonetheless, the positive control, Cl-amidine (50 µM), showed slightly higher inhibitory activities as it managed to suppress citrulline production to a reduced concentration of 9.49 ± 0.65 mM.

### 3.3 Molecular docking of selected ligands against PPAD

In order to investigate the binding interactions of the phytocompounds postulated to be present in CCM (mangiferin, vismiaquinone A, δ-tocotrienol, α-tocotrienol and canophyllol) in the binding site of PPAD crystal structure (PDB ID: 4YTB), molecular docking was performed using Schrödinger modeling software (Maestro version 12.7).

**Fig. 5:**
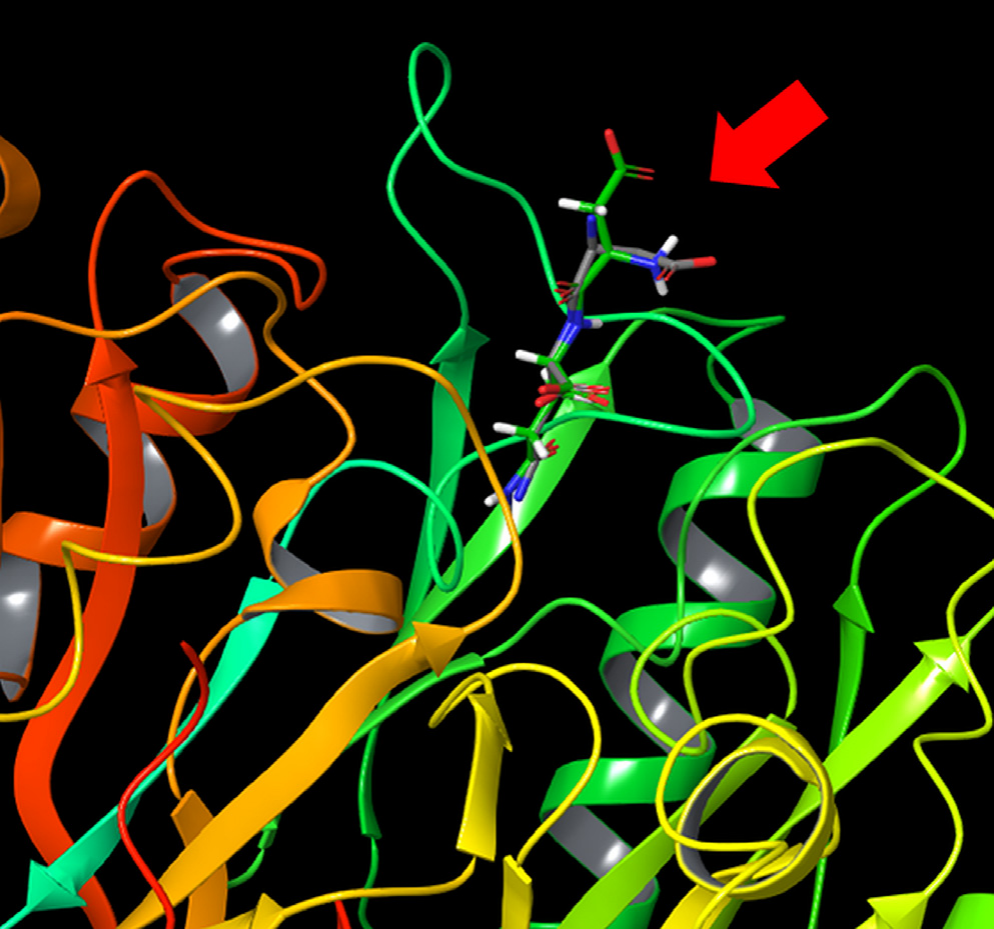
The superimposition of re-docked Asp-Gln dipeptide (in green) with co-crystallized dipeptide (in grey) in the binding pocket of PPAD (4YTB).

The dipeptide reference ligand (aspartic acid–glutamine, a co-crystallized dipeptide in 4YTB) was re-docked into the binding pocket of PPAD structure to validate the docking protocols employed. The binding mode of the re-docked ligand was then compared to that of the co-crystallized ligand; both ligands were found well superimposed onto each other along the dipeptide backbone and had similar binding modes within the binding pocket of 4YTB **(**Fig. 5**)** This inferred that the Glide docking program could well predict the binding pose as well as the interactions between ligand and adjacent residues in the binding site of 4YTB.

**Fig. 6:**
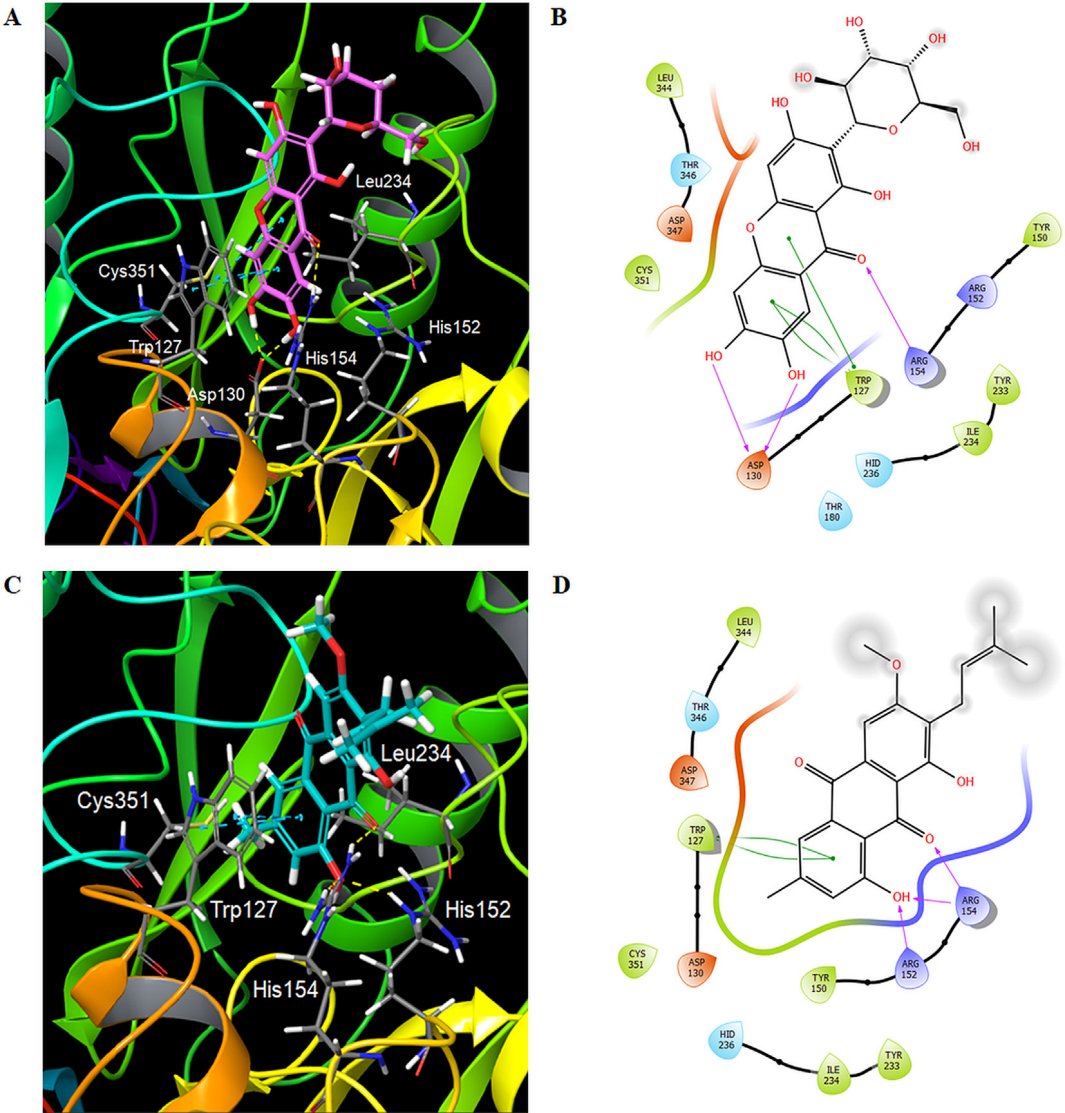
Binding orientations and interactions of mangiferin (highlighted in magenta) (A) vismiaquinone A (highlighted in blue) (C) in the binding pocket of PPAD (4YTB). Blue dotted line: pi-pi stacking; yellow dotted line: hydrogen bonding. 2D ligand interaction diagrams of mangiferin (B) and vismiaquinone A (D) depicting interactions between the ligand and binding site residues. Green line: pi-pi stacking; purple line: hydrogen bonding.

Upon docking, mangiferin (**1**) and vismiaquinone A (**2**) were found situated within the binding cavity surrounded by important residues, such as Asp130, His236, Asn297, Cys351, Arg152 and Arg154 (Montgomery et al., 2016). Both ligands engaged in hydrogen bonding with Asp130, Arg152 and Arg154, while pi-pi stacking interaction was observed with Trp127. In addition, the ligands also formed interactions with hydrophobic residues, such as Tyr150, Tyr233, Ile234, Leu344 and Cys351 and polar residues, like His236 and Thr346 (Fig. 6). However, δ-tocotrienol (**3**), α-tocotrienol (**4**) and canophyllol (**5**) were unable to fit well into the narrow binding pocket of 4YTB due to the relatively bulky side chain and scaffold of these ligands. The docking scores of the ligands are tabulated in Table 1.

**Table 1:** Docking scores of the ligands based upon Glide SP protocol.

Compounds	Docking Scores
Mangiferin	−3.776
Vismiaquinone A	−4.017
δ-Tocotrienol	−3.122
α-Tocotrienol	−2.723
Canophyllol	−2.900

## 4 Discussions

Phenolic compound is the largest class of secondary metabolites present in plants. This group of phytochemicals are diverse in structures, from as simple as phenolic acids to complex polyphenols such as flavonoids (Cheynier, 2012). The methanolic leaves extract of *Cratoxylum cochinchinense* (CCM) evaluated was found to be rich in both phenolics as well as flavonoid contents. Notably, phenolic compounds such as mangiferin (Tang et al., 2004), vismiaquinone A, δ-tocotrienol and α-tocotrienol (Chailap et al., 2017) were reported present in the leaves extract. In addition, a triterpene (canophyllol) was also identified in this part of the plant (Lv et al., 2019). Since previous studies had shown strong correlation between phenolic compounds and antioxidative activities, this extract was also tested for radical scavenging potentials as well as reducing power and metal chelating properties. Interestingly, hydroalcoholic leaves extract of *Cratoxylum cochinchinense* was not reported for these bioactivities before. Nevertheless, the widely commercialized Huang Niu Cha (Yellow Cow Wood Tea) made from the leaves of this plant showed presence of phenolic compounds and DPPH radical scavenging activities, higher than other traditional tea beverages (Bi et al., 2016).

DPPH free radical scavenging activity assay works based on the mechanism of single electron transfer reaction. DPPH is a free radical that is unstable because of the presence of unpaired electron. It is shown purple in colour until hydrogen atoms are donated to the free radicals by antioxidants, reducing them into neutral form of DPPH which are decolorized into yellow (Huyut et al., 2017). Another type of radical scavenging assay utilizes the ABTS radical cation, a stable compound that is soluble in both aqueous and organic solvents. Therefore, hydrophilic and lipophilic compounds that contribute to the antioxidant potential can be determined using this assay (Boligon, 2014). In both assays, CCM displayed significantly stronger radical scavenging activities than the positive controls used; ascorbic acid (DPPH radical scavenging assay) **(**Fig. 2 **(A))** and Trolox (ABTS radical scavenging assay) **(**Fig. 2 **(B))**. This clearly indicated that CCM is an effective free radical scavenger with the ability to prevent free radical formation, which can protect cells from being damaged by oxidative stress.

The ferric reducing antioxidant power (FRAP) assay was conducted using the method as described by Benzie and Strain (1999). In this assay, reduction of ferric ions shows a change in colour when the ions are being reduced by redox active and electron-donating compounds such as antioxidants. Ferric ions in the FRAP working reagent will form a complex with 2,4,6-Tripyridyl-S-triazine (TPTZ) which makes the solution turns pale yellow. When Fe^3+^-TPTZ complex is being reduced to Fe^2+^-TPTZ complex with the addition of antioxidants, the reaction mixture will turn from yellow to blue (Benzie and Devaki, 2017). In our study it was found that CCM had a FRAP value of 99.33 ± 13.28 mg Fe(II)/g crude extract indicating that the extract should contain reducing agents to convert ferric ions into its ferrous form. The reducing potential of 223 medicinal plants in China were also examined for FRAP (Li et al., 2013) and all extracts demonstrated reducing potentials. But only a weak correlation was observed between FRAP value and total phenolic content implying other phytocompounds could be responsible for this antioxidative mechanism.

Polyphenols consist of hydroxyl and carboxyl groups that are able to bind iron and copper which indirectly prevents these metal ions from generating oxyradicals (Nićiforović et al., 2010). In this assay, ferrozine was used to form a complex with ferrous ions. The chelating agents if present in CCM would compete and interrupt the Fe^2+^- Ferrozine complexes, leading to decolourization of purple colour formed by this chemical interaction (Gupta, 2015). A number of medicinal plants had been reported to possess significant metal chelating activities. *Bauhinia kockiana* (Kock’s Bauhinia) and *Cassia surattensis* (Glossy Shower), for instance, showed chelating activities of approximately 20 to 40% at concentration of 3.4 mg/mL. At the same concentration, leaves of *Caesalpinia pulcherrima* (Peacock Flower) exhibited stronger chelating activity of around 70% (Chew et al., 2009). Nonetheless, CCM exerted slightly weaker activity of 31% when examined at a concentration of 5 mg/mL (Fig. 2 **(C)**).

**Fig. 7:**
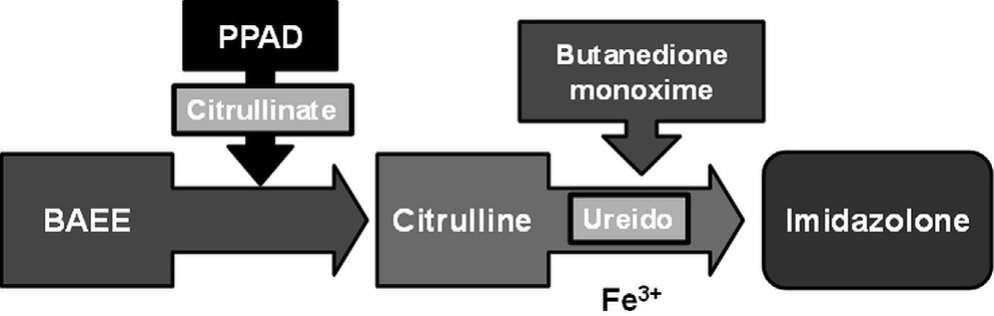
PPAD mediates the citrullination of BAEE to citrulline. Under high temperature and acidic conditions, the reaction between butanedione monoxime and the ureido groups of citrullines produces imidazolones.

In order to determine the inhibitory potential of the antioxidant-rich *Cratoxylum cochinchinense* towards *Porphyromonas gingivalis* peptidyl arginine deiminase (PPAD), a citrullination assay was performed. This colorimetric test measures the reaction between butanedione monoxime and citrulline under acidic conditions. As PPAD catalyzes the citrullination of BAEE, the guanidine group present in BAEE is converted into an ureido group (Fig. 7). These ureido groups present in the resulting citrullines react with butanedione monoxime under catalysis of ferric ions (derived from the Redox reagent) to produce colorizing compounds known as imizadolones, giving rise to a rapid colour development that can be detected spectrophotometrically (Slade et al., 2014). Citrulline content is then determined by comparing the absorbance of biological samples against a prepared citrulline standard curve (Teo et al., 2012).

A dose dependent inhibition of the citrullination activity of the recombinant PPAD in the cell lysate was observed when samples were treated with CCM. The extract was able to reduce the citrulline concentration significantly better than untreated control. However, its inhibitory activity was not as strong as the positive control used, Cl-amidine (50 µM) **(**Fig. 4**)**. Cl-amidine or also known as N-α-benzoyl-N5-(chloro-iminoethyl)- L -ornithine amide is a non-selective inhibitor of human peptidylarginine deiminases (PADs). It functions by modifying a cysteine residue present in the active site of these enzymes thus irreversibly inactivating their catalytic activities (Luo et al., 2006). It is also discovered by Bereta et al. (2019) that this molecule is also able to block the citrullination of bacterial PAD as well, thus, justifying the use of Cl-amidine as positive control.

We postulated that the phytochemicals reportedly present in the leaves extracts of *Cratoxylum cochinchinense* such as mangiferin (Tang et al., 2004), vismiaquinone A, δ-tocotrienol and α-tocotrienol (Chailap et al., 2017) as well as canophyllol (Lv et al., 2019) could be responsible for the observed PPAD inhibitory effects. Therefore, these secondary metabolites were subsequently used as ligands in the simulated docking against PPAD to evaluate their binding orientations and interactions in the binding pocket of the protein.

Based on the docking results, both mangiferin (1) and vismiaquinone A (2) were well fitted within the binding site cavity. For mangiferin, two of hydroxyl groups on the phenyl ring formed hydrogen bonding with Asp130, and another hydrogen bonding was observed with Arg154 via adjacent carbonyl oxygen atom. Pi-pi stacking interaction was seen between the aromatic rings of ligand and Trp127. The non-polar moieties of ligand were surrounded by Tyr150, Tyr233, Ile234, Leu344 and Cys351. Polar residues, His236 and Thr346 were also found to interact with the polar group of the ligand, such as hydroxyl groups and oxygen atom on the xanthone nucleus (Fig. 6 **(A)** and **(B)**). In vismiaquinone A, hydroxyl group on the phenyl ring engaged two hydrogen bonding, each with Asp152 and Asp154; another hydrogen bonding was formed with Arg154 through the carbonyl oxygen atom in vicinity. Likewise, pi-pi stacking interaction was found between the phenyl ring of ligand and Trp127. The non-polar moieties and polar group of the ligand were also delineated by the same residues, namely Tyr150, Tyr233, Ile234, Leu344, Cys351 and His236, Thr346, respectively (Fig. 6 **(C)** and **(D)**). Among the residues, Asp130, Arg152, Arg154, His236 and Cys351 have been shown to play the important role in ligand recognition and binding (Montgomery et al., 2016, Goulas et al., 2015).

On the other hand, the presence of long alkenyl and alkyl chain in δ-tocotrienol (3) and α-tocotrienol (4), respectively had impeded the entry of these compounds into the narrow active site cleft of 4YTB. Similarly, the bulky and rigid fused ring structure of canophyllol (5) was found unable to fit into the binding pocket of 4YTB. Additionally, the docking scores of the ligands (Table 1) were also examined. Such scores are the scoring function resulting from the evaluated docking pose when a ligand is bound inside the binding cavity of a target protein; more negative values indicate tighter binders (Pantsar and Poso, 2018). Consistently, both mangiferin and vismiaquinone A showed higher docking scores than that of δ-tocotrienol, α-tocotrienol and canophyllol, suggesting that both could bind comparatively better in the binding pocket of PPAD. Hence, it is deduced that mangiferin and vismiaquinone A are the potential phytocompounds that contribute to the inhibitory activities against PPAD as observed from the tested CCM leaves extract.

## 5 Conclusion

The findings in the present study highlighted the efficacy of *Cratoxylum cochinchinense* methanolic leaves extract (CCM) in inhibiting the citrullination process of *Porphyromonas gingivalis* peptidylarginine deiminase (PPAD). Since citrullination is key to the microbe’s virulence mechanism and the main trigger of many systemic diseases, the inhibitory potential of CCM ultimately makes it a promising therapeutic agent for periodontitis as well as diseases like rheumatoid arthritis. Furthermore, the plant not only shows potential as inhibitor of PPAD but also acts as a capable antioxidant based on the radical scavenging, metal chelating and reducing power activities. From the molecular docking study, phenolic compounds; mangiferin and vismiaquinone A as reported in *Cratoxylum cochinchinense* leaves are found to interact with important amino acid residues of the PPAD protein suggesting their inhibitory effects on the PPAD. Therefore, further investigations on these two compounds shall be conducted to validate their bioactivities as well as to investigate their effects *in vivo*.

## Declaration of Competing Interest

The authors declare that they have no known competing financial interests or personal relationships that could have appeared to influence the work reported in this paper.
